# Influence of the Spent Coffee Ground-to-Ammonium Lignosulfonate Ratio on the Performance of Wood-Fiber Biocomposites

**DOI:** 10.3390/ma19173748

**Published:** 2026-09-03

**Authors:** Viktor Savov, Petar Antov, Alexandrina Kostadinova-Slaveva, Ekaterina Todorova, Jansu Yusein, Georgi Ivanov, Viktoria Dudeva, Konstantinos Ninikas, Stoyko Petrin, Lee Seng Hua

**Affiliations:** 1 Faculty of Forest Industry, University of Forestry, 1797 Sofia, Bulgaria; yusein@ltu.bg (J.Y.); georgi_ivanov@ltu.bg (G.I.); v.dudeva@ltu.bg (V.D.); 2 Center of Competence “Clean Technologies for a Sustainable Environment—Water, Waste, Energy for a Circular Economy”, 1407 Sofia, Bulgaria; aslaveva@ltu.bg (A.K.-S.); etodorova@ltu.bg (E.T.); 3 Faculty of Ecology and Landscape Architecture, University of Forestry, 1797 Sofia, Bulgaria; 4 Department of Forestry, Wood Sciences & Design, School of Technology, University of Thessaly, 43100 Karditsa, Greece; kninikas@uth.gr; 5 Faculty of Chemical Technologies, University of Chemical Technology and Metallurgy, 1757 Sofia, Bulgaria; stpetrin@uctm.edu; 6 Department of Wood Industry, Faculty of Applied Sciences, Universiti Teknologi MARA Pahang Branch Jengka Campus, Bandar Tun Abdul Razak 26400, Pahang, Malaysia; leesenghua@uitm.edu.my; 7 Institute for Infrastructure Engineering and Sustainable Management (IIESM), Universiti Teknologi MARA, Shah Alam 40450, Selangor, Malaysia

**Keywords:** wood-fiber biocomposites, spent coffee grounds, ammonium lignosulfonate, bio-based binder, physical and mechanical properties, thermal analysis, circular bioeconomy

## Abstract

Spent coffee grounds (SCGs) and ammonium lignosulfonate (ALS) are promising renewable constituents for wood-fiber biocomposites, but the effect of their relative proportion within the binder phase remains insufficiently understood. This preliminary study evaluated wood-fiber panels produced at a constant dry-matter matrix-to-fiber ratio of 50:50 while varying the SCG:ALS ratio from 70:30 to 30:70. The panels were hot-pressed and characterized for their physical and mechanical properties, while the corresponding SCG–ALS matrix formulations were evaluated by simultaneous TGA–DSC analysis. Increasing the SCG fraction generally improved dimensional stability and mechanical performance, whereas formulations containing 50% or more ALS showed a marked deterioration in water resistance. The SCG-rich 70:30 formulation provided the highest bending performance, with MOE and MOR values of 2156.5 and 12.83 N·mm^−2^, respectively. Thermal analysis revealed ratio-dependent changes in mass loss and in the principal heat-flow event, which occurred between approximately 113 and 136 °C, confirming that the SCG:ALS ratio altered the early thermal behavior of the binder system. Overall, the SCG:ALS ratio was identified as a key formulation parameter, with SCG-rich systems providing the most favorable balance between mechanical performance, dimensional stability, and internal cohesion.

## 1. Introduction

Wood-based panels are essential engineered materials for furniture, interior products, and construction, but their continued development is increasingly shaped by the need to reduce fossil-resource consumption, formaldehyde emissions, and dependence on virgin lignocellulosic raw materials. Recent advances in bio-based adhesives and formaldehyde-free panel technologies demonstrate considerable progress while also confirming that renewable adhesive systems must satisfy demanding requirements related to reactivity, water resistance, mechanical performance, storage stability, and compatibility with industrial hot pressing [1]. In parallel, circularity-oriented strategies for wood-based panel manufacturing increasingly emphasize the use of secondary fibers, agricultural residues, and industrial side streams as functional raw materials rather than low-value waste [2]. Bio-based adhesives derived from proteins, carbohydrates, tannins, lignin, and other renewable resources are therefore being investigated as complete substitutes for conventional resins or as components of hybrid bonding systems [3].

Commercial wood composites are still predominantly manufactured with urea–formaldehyde, melamine–urea–formaldehyde, phenol–formaldehyde, and polymeric isocyanate binders because of their high bonding efficiency and compatibility with rapid industrial production [4]. Urea–formaldehyde resins are particularly important because of their relatively low cost, water solubility, short curing time, and high dry bonding strength [5]. Nevertheless, increasingly stringent emission requirements continue to stimulate research on ultra-low-formaldehyde and no-added-formaldehyde systems [6]. Several reviews have concluded that full replacement of conventional resins remains challenging because many renewable binders exhibit lower reactivity, higher viscosity, greater moisture sensitivity, and more variable chemical composition [7]. Consequently, successful bio-based adhesive development depends not only on renewable content but also on the establishment of complementary interactions among the individual binder constituents [8]. The selection and proportioning of these constituents are therefore critical for achieving a suitable balance between processing behavior, interfacial adhesion, dimensional stability, and mechanical performance [9].

Spent coffee grounds (SCGs) are a high-volume agri-food residue with considerable potential for material valorization. Their current applications include fuels, adsorbents, soil amendments, chemical feedstocks, and polymer-composite fillers [10]. SCGs are generated after hot-water extraction of roasted coffee and retain substantial amounts of lignin, cellulose, hemicelluloses, proteins, lipids, polyphenolic substances, residual carbohydrates, and mineral components [11]. Their exact chemical composition is influenced by coffee variety, roasting conditions, grinding, extraction procedure, and post-brewing treatment [12]. Structural analyses have confirmed that SCGs are heterogeneous lignocellulosic particles rather than chemically uniform fillers [13]. From an environmental perspective, their relatively high moisture content and susceptibility to microbial deterioration complicate storage and transport, while material valorization offers an alternative to disposal and is consistent with circular bioeconomy strategies for converting SCG waste into value-added products [14,15,16].

The complex composition of SCGs has led to their incorporation into numerous polymeric systems. Their use as renewable composite fillers has demonstrated potential for improving stiffness, natural coloration, and renewable carbon content, although particle agglomeration and weak interfacial adhesion may reduce strength at high loading levels [17]. Across polypropylene, PLA, PLA–starch–sucrose, paper-pulp, PHBV, and related biodegradable composite systems, SCG performance has been shown to depend strongly on surface treatment, filler loading, particle size, dispersion, and interfacial compatibility [18,19,20,21,22,23,24,25,26].

Lignin and lignin-derived products represent another major class of renewable polymeric raw materials. Industrial lignin streams have considerable potential for composites, plastics, coatings, and adhesives, but their properties depend strongly on botanical source and isolation process [27]. Lignin is characterized by a complex, three-dimensional, crosslinked aromatic structure containing phenolic and aliphatic hydroxyl groups, methoxy groups, and other reactive functionalities that enable hydrogen bonding, interfacial interactions, chemical modification, and thermally induced condensation and crosslinking reactions [28]. Its incorporation into polymer systems may provide rigidity and other functional benefits, while chemical modification can further enhance lignin reactivity and compatibility, although at the cost of additional formulation and processing complexity [29,30].

The use of lignin in wood adhesives has been widely investigated because of its structural similarity to phenolic compounds and its natural affinity for lignocellulosic surfaces. However, unmodified technical lignins often show limited reactivity and may require activation, crosslinking, or combination with other adhesive constituents; lignin present in agricultural residues can also contribute to bonding, and oxidative modification may improve adhesive reactivity [31,32,33].

Lignosulfonates are water-soluble technical lignin derivatives generated primarily as by-products of sulfite pulping. The introduction of hydrophilic sulfonate groups into their heterogeneous aromatic macromolecular structure provides excellent water solubility, dispersing capacity, and affinity for lignocellulosic surfaces. However, these functional groups also increase hygroscopicity and water sensitivity, which may adversely affect the dimensional stability and moisture resistance of lignosulfonate-bonded composites. Ammonium lignosulfonate (ALS) has been investigated as a renewable binder for particleboard manufacture in combination with polymeric methylene diphenyl diisocyanate (pMDI) and furfuryl alcohol, demonstrating that enhanced crosslinking and interfacial interactions can substantially improve bonding performance and moisture resistance [34]. Related studies using lignosulfonates in urea–formaldehyde systems or as binders for recycled wood fibers further confirm their potential, but also their dependence on reactive co-binders and suitable processing conditions [35,36]. Comparisons among lignosulfonate types and formulations show that molecular structure, counter-ion type, solid content, curing behavior, substitution level, and interactions with co-binders strongly influence mechanical performance, moisture resistance, and formaldehyde-related properties [37,38,39,40,41,42,43].

Combining SCGs and ALS may provide complementary functions in a wood-fiber biocomposite. ALS can form a water-soluble phase that spreads over wood fibers and SCG particles, while SCGs contribute lignocellulosic particles, residual carbohydrates, lignin, lipids, and thermally active extractives. Hydrogen bonding among lignin-derived hydroxyl groups, SCG carbohydrates, and cellulose surfaces may contribute to interfacial adhesion [44]. More broadly, bonding in natural-binder systems can involve mechanical interlocking, secondary interactions, molecular rearrangement, thermally induced reactions, densification, redistribution of soluble constituents, and partial self-bonding during hot pressing [45,46,47].

The thermal behavior of the SCG–ALS matrix is inherently complex because neither constituent represents a chemically homogeneous polymer. The principal lignocellulosic components of SCGs, i.e., hemicelluloses, cellulose, and lignin, undergo degradation over broad, partially overlapping temperature ranges, although their characteristic decomposition profiles remain distinguishable [48]. Variations in lignin content may further influence the mass-loss rate, thermal-degradation pathway, char formation, and distribution of volatile decomposition products [49]. Consequently, simultaneous mass-loss and heat-flow events in the SCG–ALS matrix should be interpreted as overlapping dehydration, volatilization, and early degradation processes rather than as simple melting or glass-transition phenomena.

In our preceding study, wood-fiber biocomposites were produced using SCGs and ALS in a fixed 1:1 ratio, while their combined content was varied between 40 and 75% relative to the oven-dry fiber mass [50]. That investigation established the technical feasibility of the formaldehyde-free SCG–ALS bonding concept and demonstrated that total matrix content strongly affected density, water absorption, thickness swelling, modulus of elasticity, bending strength, internal bond strength, and thermogravimetric behavior. The formulation containing a combined SCG–ALS amount of approximately 50% relative to the oven-dry wood-fiber mass provided the most favorable overall mechanical performance, whereas higher total matrix contents generally increased moisture sensitivity.

A subsequent investigation extended the same material platform toward aesthetic characteristics and exploratory end-of-life behavior [51]. The SCG and ALS fractions were again maintained at equal proportions, while the total matrix content was varied. That study demonstrated composition-dependent changes in panel appearance and confirmed visible microbial colonization and progressive surface degradation during exploratory composting. Together, the two preceding investigations established the technical feasibility and several key performance characteristics of the wood-fiber/SCG/ALS system, but neither isolated the effect of the internal SCG:ALS composition.

However, both preceding investigations changed the total SCG–ALS content while maintaining a fixed SCG:ALS ratio of 1:1. Therefore, they did not isolate the contribution of the internal matrix composition. It remains unclear whether the previously identified optimum resulted from the selected total matrix loading, from the equal balance between SCGs and ALS, or from an interaction between these two factors. Published studies on SCG composites predominantly treat SCGs as particles dispersed in a pre-existing polymer matrix, whereas lignosulfonate studies generally consider lignosulfonates as resin modifiers, partial adhesive substitutes, or binders combined with reactive crosslinkers. The behavior of systems in which SCGs and ALS jointly constitute the formaldehyde-free matrix of a wood-fiber composite remains insufficiently understood.

The resulting problem statement is that rational optimization of the wood-fiber/SCG/ALS biocomposite is restricted when the total matrix amount and its internal composition are varied simultaneously. Under such conditions, changes in mechanical performance, water resistance, dimensional stability, and thermal response cannot be attributed specifically to the coffee-derived or lignosulfonate-derived fraction. The corresponding research gap is the absence of a systematic composition–property study in which the total matrix-to-fiber ratio is held constant, while only the SCG:ALS ratio is varied. This distinction is particularly important because broad and overlapping thermal processes are characteristic of lignin-containing biocomposites and may provide additional evidence concerning matrix consolidation and thermal stability [52].

Accordingly, the aim of the present study was to determine the influence of the SCG:ALS ratio on the physical, mechanical, and thermal performance of wood-fiber biocomposites at a constant total matrix content. Five SCG:ALS ratios, ranging from 70:30 to 30:70, were evaluated under comparable panel-manufacturing conditions. The resulting composites were characterized in terms of density, water absorption, thickness swelling, modulus of elasticity, bending strength, internal bond strength, and simultaneous TGA–DSC behavior. It was hypothesized that increasing the ALS fraction would improve matrix continuity but could increase hygroscopicity and moisture sensitivity, whereas increasing the SCG fraction could reduce the amount of continuous water-soluble binder while contributing a more particulate and potentially less hygroscopic matrix structure.

The novelty and significance of the present study lie in isolating the internal composition of the SCG–ALS matrix as an independent formulation variable. Unlike the preceding investigations, which optimized the total amount of a fixed 1:1 SCG–ALS matrix, the present work maintains a constant matrix-to-fiber ratio and systematically varies only the relative amounts of SCG and ALS. This design enables a direct composition–property relationship to be established and allows the respective roles of the particulate coffee-derived fraction and the soluble lignosulfonate phase to be distinguished more clearly. From an application perspective, identifying a favorable SCG:ALS formulation window provides a rational basis for the further development of formaldehyde-free wood-fiber biocomposites that valorize both an agri-food residue and an industrial lignin-derived side stream.

## 2. Materials and Methods

### 2.1. Raw Materials

Industrial thermomechanical wood fibers, spent coffee grounds (SCGs), and commercial ammonium lignosulfonate (ALS) were used for panel production. The raw materials and their preparation were consistent with those used in our previous studies [50,51].

The wood fibers were supplied by Kronospan Bulgaria EOOD, Veliko Tarnovo, Bulgaria, and had an initial moisture content of approximately 11%. The SCGs were collected from local coffee shops, dried at approximately 20 °C to a moisture content of 9.8%, and stored in sealed containers until use. Commercial ammonium lignosulfonate D-947L, supplied by Borregaard, Sarpsborg, Norway, was used as an aqueous solution containing 48.6% solids.

### 2.2. Experimental Design and Panel Manufacture

**Figure 1:**
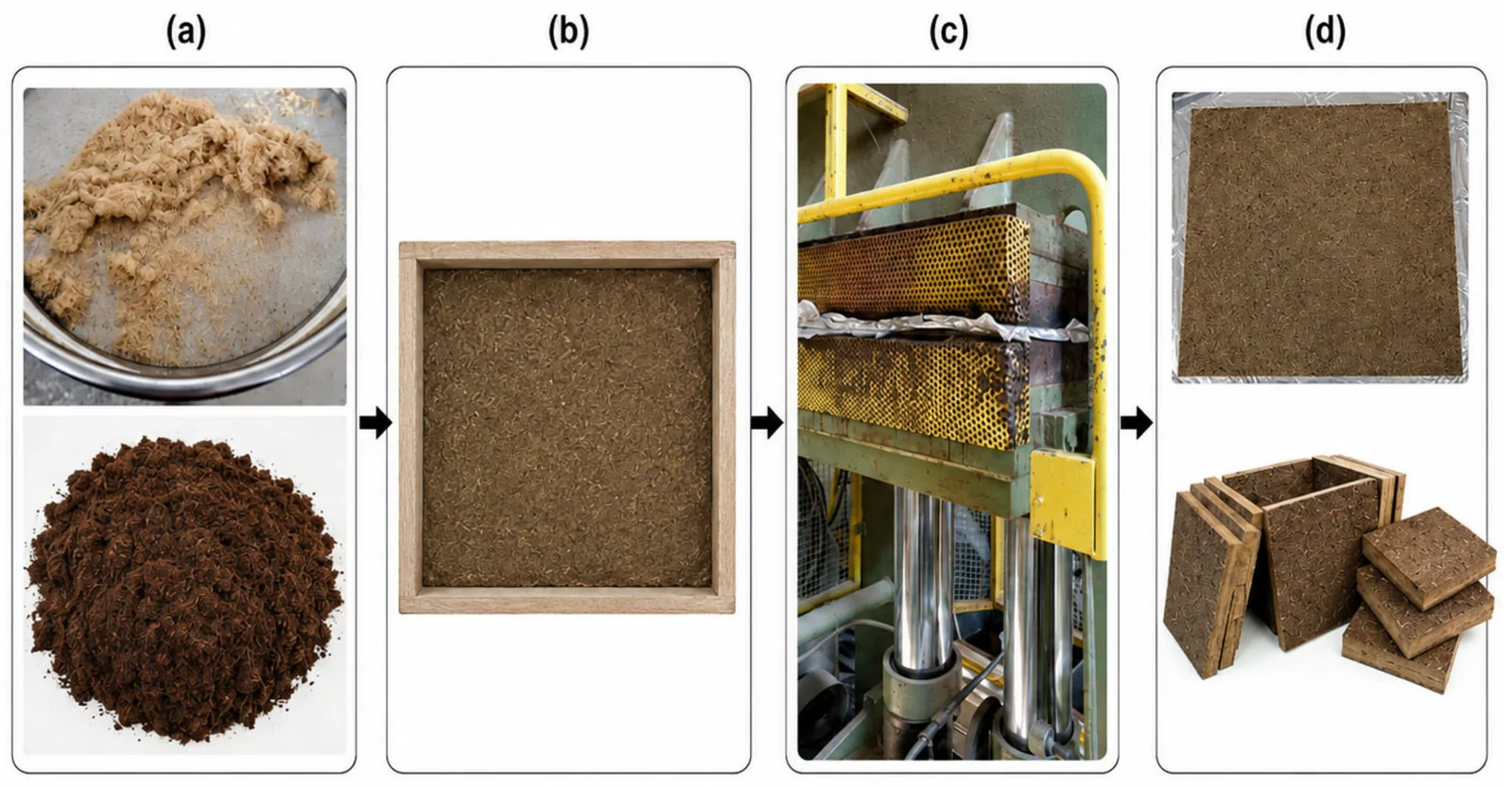
Schematic illustration of the main stages in the production of SCG–ALS-bonded wood-fiber biocomposites: (**a**) raw materials; (**b**) formed mat; (**c**) hot pressing; and (**d**) produced panel and representative cut specimens.

The experimental design was based on five SCG:ALS ratios, while the total matrix content, suspension concentration, target panel density, and manufacturing conditions were kept constant. The equal SCG:ALS ratio used in our preceding studies was retained as the central formulation, while the symmetric range from 70:30 to 30:70 was selected to determine the effect of shifting the matrix composition toward either constituent under otherwise constant manufacturing conditions [50,51]. The dry-matter ratio between the SCG–ALS matrix and the wood fibers was 50:50, and the total solid concentration of the matrix suspension was adjusted to 50 wt.%. This solid concentration was maintained for all formulations to provide comparable mixing and distribution conditions while limiting the amount of water that had to be removed during hot pressing since excessive moisture was previously associated with reduced dimensional stability and internal bond strength [50]. The investigated SCG dry-matter ratios of 70:30, 60:40, 50:50, 40:60, and 30:70 were designated as SCG70–ALS30, SCG60–ALS40, SCG50–ALS50, SCG40–ALS60, and SCG30–ALS70, respectively. In these designations, the numbers indicate the mass fractions (%) of SCG and ALS in the dry matrix solids. The main stages of the biocomposite manufacturing process are schematically illustrated in Figure 1.

For each formulation, SCGs and ALS were mixed in the required dry-matter ratio, and water was added to obtain a suspension containing 50 wt.% solids. The suspension was gradually added to the wood fibers and mechanically mixed for 10 min until a visually homogeneous furnish was obtained. No synthetic resin, crosslinker, catalyst, or hydrophobizing agent was added.

The furnish was manually formed into a single-layer mat and hot pressed in a laboratory hydraulic press, type PMC ST 100 (Manni, Italy). One panel with nominal dimensions of 400 mm × 400 mm × 6 mm and a target density of 750 kg·m^−3^ was produced for each formulation. The target density of 750 kg·m^−3^ was retained from the preceding investigations because it corresponds to the typical density range of conventional MDF panels and enables direct comparison with the previously developed SCG–ALS wood-fiber composites [50,51].

Hot pressing was conducted at 150 °C for a total of 16 min using a two-stage pressure regime: 1.8 MPa for 1 min, followed by 0.6 MPa for 15 min. The pressing temperature and pressure schedule were selected on the basis of the previously established processing window for this material system. The initial high-pressure stage promoted rapid mat consolidation, whereas the prolonged lower-pressure stage facilitated matrix redistribution and moisture release while avoiding unnecessarily severe thermal exposure of the lignocellulosic components [50]. After hot pressing, the panels were cooled under restraint in a cold press at 0.2 MPa until reaching approximately room temperature. The panels were subsequently conditioned in a FAITHFUL climatic chamber (Faithful, model FPG250, Cangzhou, China) at 20 ± 2 °C and 65 ± 5% relative humidity until constant mass.

### 2.3. Physical and Mechanical Characterization

Test specimens were prepared according to EN 326-1 [53], and their dimensions were verified according to EN 325 [54]. Eight specimens were tested for each formulation and property.

Density was determined according to EN 323 [55] using specimens with dimensions of 50 mm × 50 mm. Water absorption and thickness swelling were evaluated after 24 h immersion in water at 20 ± 1 °C. Thickness swelling was determined according to EN 317 [56], while water absorption was calculated gravimetrically using the same specimens.

The modulus of elasticity and bending strength were determined by three-point bending according to EN 310 [57] using a WDW-50E universal testing machine (HST, Jinan, China). The bending specimens measured 50 mm × 160 mm, the support span was 120 mm, and the crosshead speed was 5 mm·min^−1^.

Internal bond strength was determined according to EN 311 [58] using 50 mm × 50 mm specimens bonded to metal loading blocks and loaded perpendicular to the panel plane until failure.

### 2.4. Simultaneous Thermal Analysis of the SCG–ALS Matrix Formulations

Simultaneous thermogravimetric and differential scanning calorimetry analysis was performed on the five SCG–ALS matrix formulations before their incorporation into the wood fibers. Therefore, the thermal results characterize the matrix mixtures rather than the finished panels. The matrix formulations were analyzed in their moisture-containing processing state, without additional pre-drying before STA, in order to retain the thermal effects associated with moisture release and other early transformations occurring during heating.

The measurements were performed using an HS-TGA-103 simultaneous thermal analyzer (Yantai Stark Instrument Co., Ltd., Shandong, China). One measurement was conducted for each SCG:ALS ratio in air using open platinum crucibles and a nominal heating rate of 5 °C min^−1^.

The samples were heated from approximately room temperature to 100 °C, held isothermally at 100 °C for approximately 4.9 min to promote the removal of free and weakly bound water, and subsequently heated to approximately 238 °C. TG, DTG, and heat-flow signals were recorded.

For comparative evaluation, the heat-flow curves were baseline-corrected after the 100 °C isothermal holding stage and normalized to the sample mass recorded at the end of this stage. The instrumental heat-flow sign convention was retained. The thermal measurements were used for comparative evaluation of mass loss, mass-loss rate, and the principal heat-flow events of the different SCG–ALS formulations. The investigated temperature range was selected primarily to characterize processing-relevant early thermal events, including moisture release, volatilization, and the initial thermal transformations of the SCG–ALS matrix occurring around and above the hot-pressing temperature, rather than to determine its complete high-temperature degradation profile.

### 2.5. Data Processing

For each physical and mechanical property, the arithmetic mean, standard deviation, and coefficient of variation were calculated from the eight tested specimens. For visualization of the flexural response, the individual stress–strain curves were linearly interpolated to a common strain scale and averaged for each formulation; mean values were retained while at least six of the eight specimens contributed measured data at the corresponding strain level.

Since one laboratory panel was produced for each formulation, the specimens represented within-panel subsamples rather than independent panel-production replicates. Accordingly, the results were evaluated descriptively as composition-dependent trends under the applied laboratory conditions. The thermal analysis was also interpreted comparatively because one measurement was available for each matrix formulation.

## 3. Results and Discussion

### 3.1. Panel Density and Formulation-Dependent Consolidation

**Figure 2:**
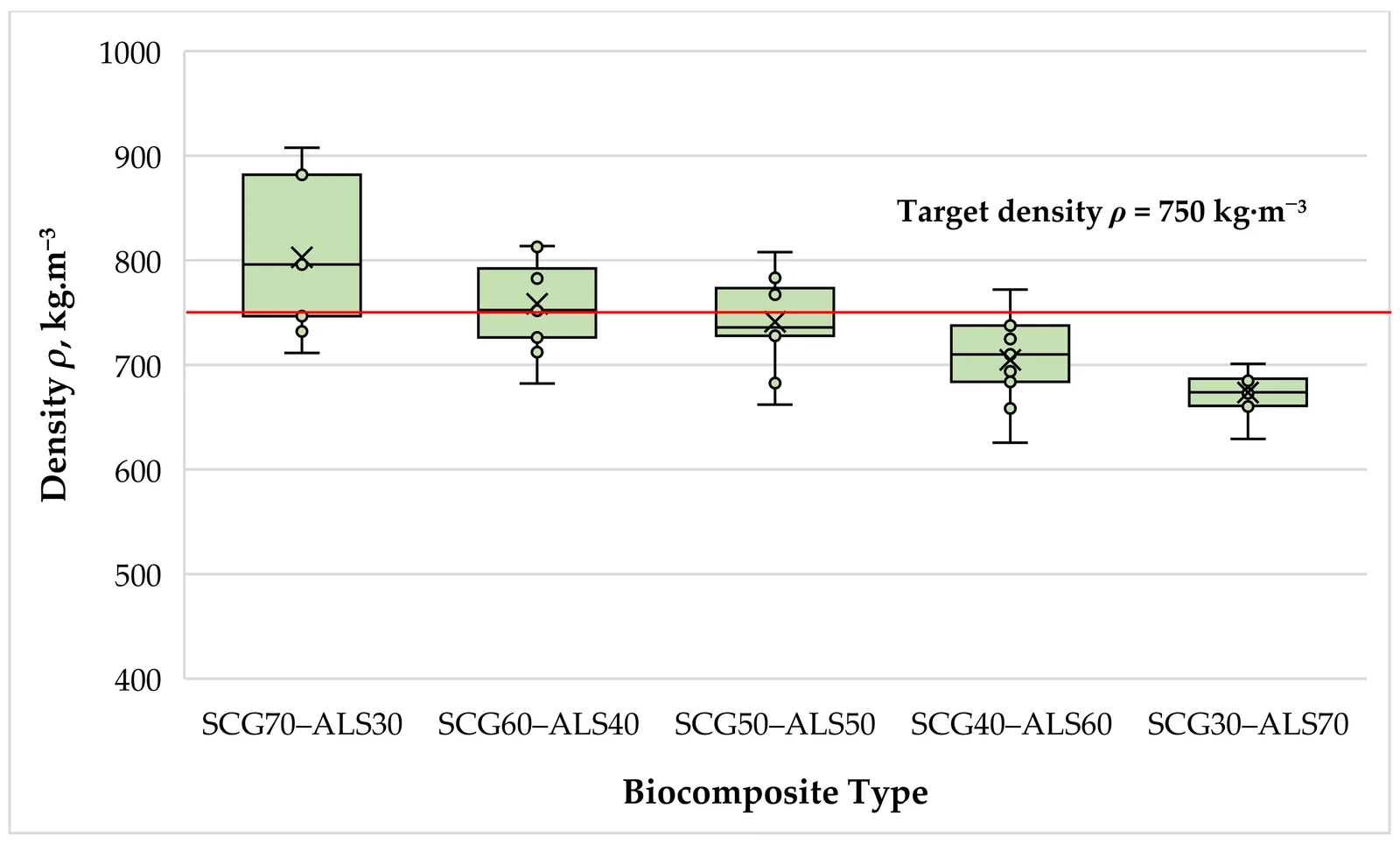
Density distribution of the wood-fiber biocomposite panels produced with different SCG:ALS ratios. The boxes indicate the interquartile range, the horizontal line and cross within each box represent the median and arithmetic mean, respectively, and the whiskers indicate the range of non-outlying values. The horizontal red line indicates the target density of 750 kg m^−3^; n=8 within-panel specimens per formulation.

Although all panels were manufactured using the same nominal dimensions, target density, matrix-to-fiber ratio, suspension concentration, and pressing schedule, the achieved density was affected by the internal composition of the SCG–ALS matrix. As shown in Figure 2, the mean panel density decreased progressively from 814.3 kg·m^−3^ for SCG70–ALS30 to 679.3 kg·m^−3^ for SCG30–ALS70. The SCG60–ALS40 and SCG50–ALS50 formulations remained comparatively close to the target density of 750 kg·m^−3^, whereas the two formulations containing 60 and 70% ALS were below the target value.

The systematic decrease in density suggests that the SCG:ALS ratio affected furnish consolidation and post-press thickness recovery. SCGs constitute a heterogeneous particulate material containing lignin, carbohydrates, proteins, lipids, and mineral components [10,11,12,13]. Their solid particles may contribute to more efficient packing of the fiber network and restrict post-press spring-back. By contrast, ALS forms a water-soluble and comparatively mobile phase. Increasing its proportion increased the share of this continuous soluble phase within the matrix, although the total solid concentration and, consequently, the overall water content of the matrix suspension were maintained constant. A higher ALS proportion may therefore improve spreading over the fibers and enhance matrix mobility during pressing while also producing a more hydrophilic and readily plasticized matrix structure.

The consolidation of lignocellulosic panels bonded with natural or self-binding systems involves several simultaneous mechanisms, including mechanical interlocking, redistribution of soluble constituents, hydrogen bonding, thermal softening, water removal, and the establishment of intimate contact among the constituent particles and fibers [44,45,46,47]. A more mobile matrix does not necessarily produce a denser final panel because excessive moisture and steam pressure may promote local disturbance, delayed dimensional stabilization, or spring-back after pressure release. The present results therefore suggest that SCGs performed not only as a constituent of the binding system but also as a particulate component influencing mat compressibility and structural fixation.

In the preceding study, where the SCG:ALS ratio was fixed at 1:1 and only the total matrix content was varied, the mean panel densities remained within the comparatively narrow range of 735–756 kg·m^−3^ [50]. The wider density range obtained in the present work indicates that changing the internal composition of the matrix may influence consolidation differently from changing only its total amount.

Density is an important factor in the interpretation of the subsequent properties. Increased panel density generally improves interparticle contact, reduces void volume, and supports stress transfer within hot-pressed lignocellulosic structures [45,46,47]. Consequently, the favorable properties of the SCG-rich panels cannot be attributed exclusively to the chemical effect of the SCG:ALS ratio. Part of their performance may also originate from their higher achieved density. This composition–density interaction is considered further in Section 3.5.

### 3.2. Water Absorption and Thickness Swelling

**Figure 3:**
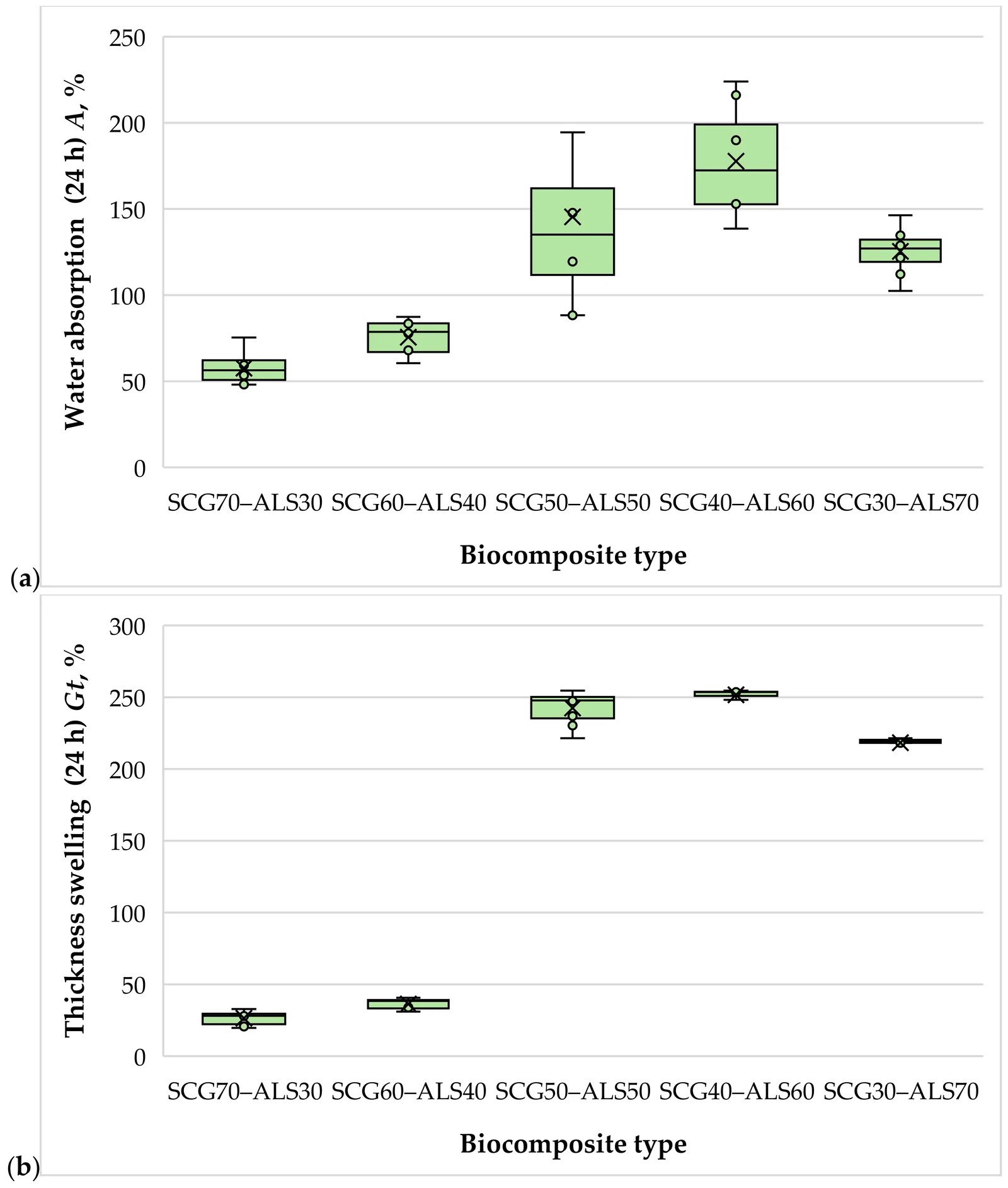
Moisture-related properties of the wood-fiber biocomposites produced with different SCG:ALS ratios after 24 h water immersion: (**a**) water absorption and (**b**) thickness swelling. The boxes indicate the interquartile range, the horizontal line and cross within each box represent the median and arithmetic mean, respectively, and the whiskers indicate the range of non-outlying values; n=8 within-panel specimens per formulation.

The SCG:ALS ratio had a pronounced influence on water absorption and thickness swelling after 24 h immersion. The lowest water absorption was recorded for SCG70–ALS30, at 57.5%, followed by SCG60–ALS40 at 75.6% (Figure 3a). Increasing the ALS proportion to 50% resulted in a sharp increase in water absorption to 145.4%, while the maximum value of 177.7% was obtained for SCG40–ALS60. A lower value of 125.3% was measured for SCG30–ALS70, although this formulation still exhibited poor dimensional stability.

Thickness swelling followed an even more distinct threshold-type response (Figure 3b). The mean value increased moderately from 26.4% for SCG70–ALS30 to 36.2% for SCG60–ALS40. When the ALS share reached 50%, however, thickness swelling increased abruptly to 242.4%. Similarly, high values of 251.7 and 218.3% were obtained for SCG40–ALS60 and SCG30–ALS70, respectively.

The results indicate the presence of a critical compositional region between 40 and 50% ALS. Below this range, water absorption and swelling increased comparatively gradually. At and above 50% ALS, the dimensional integrity of the panels deteriorated markedly.

This behavior can be related to the chemical structure of lignosulfonates. Sulfonate groups provide ALS with high water solubility and effective dispersing ability, which is beneficial during furnish preparation. The same functional groups, however, increase affinity for water and facilitate rehydration of the matrix after panel manufacture. Unmodified lignosulfonates generally show limited water resistance and comparatively low intrinsic crosslinking ability. Their successful use in wood-based panels therefore frequently involves pMDI, furfuryl alcohol, modified aminoplastic resins, or other reactive components [34,35,36,37,38,39,40,41,42,43].

Hot pressing can promote hydrogen bonding among ALS, SCG constituents, and the hydroxyl-rich surfaces of the wood fibers [44,45]. Such secondary interactions may provide substantial dry-state cohesion but remain susceptible to disruption by water. During immersion, water molecules compete for hydroxyl and sulfonate sites, plasticize the matrix, weaken interfacial interactions, and enable relaxation of compressed fibers. These combined mechanisms explain why ALS may improve furnish distribution and dry bonding at moderate proportions while impairing dimensional stability when it becomes the dominant matrix component.

Earlier studies of lignosulfonate-bonded wood composites have reported comparable limitations. Hemmilä et al. demonstrated that pMDI and furfuryl alcohol substantially improved the performance of ALS-bonded particleboards [34]. Bekhta et al. obtained improved board properties by combining lignosulfonate-containing urea–formaldehyde systems with pMDI [35], while Antov et al. showed that magnesium lignosulfonate could be used to bond recycled fibers, although the resulting properties remained strongly dependent on formulation and processing conditions [36]. Other investigations have confirmed that lignosulfonate type, counter-ion, molecular structure, substitution level, and interaction with the co-binder influence both bond development and moisture resistance [37,38,39,40,41,42,43].

The comparatively better moisture performance of the SCG-rich formulations may have several causes. Increasing SCG reduced the proportion of the continuous, water-soluble ALS phase. The particulate SCG fraction may have also contributed to improved packing and a less open internal structure. Furthermore, SCGs contain residual lipids and extractives that may locally reduce surface wettability, although their carbohydrate fraction remains hydrophilic [11,12,13,14]. Their overall influence therefore depends on the balance between particulate filling, residual hydrophobic constituents, hydrophilic polysaccharides, and interaction with ALS.

This interpretation is consistent with the broader literature on SCG-containing polymer composites. SCGs can increase renewable content and stiffness, but their influence on moisture response depends on particle dispersion, residual oil content, extraction history, and interfacial compatibility [17,18,19,20,21,22,23,24,25,26]. In composites based on SCGs and paper pulp, water sensitivity remained a major limitation despite the ability of the components to form coherent structures [22]. Studies of PLA-, polypropylene-, and PHBV-based composites similarly showed that SCGs cannot be treated as an inert filler because their heterogeneous composition affects both interfacial and moisture-related behavior [17,18,19,20,21,22,23,24,25,26].

The reduction in water absorption from 177.7% for SCG40–ALS60 to 125.3% for SCG30–ALS70 should not be interpreted as a genuine recovery of water resistance. The corresponding thickness swelling remained extremely high. In highly soluble ALS-rich systems, part of the matrix may have leached into the immersion water. Simultaneous water uptake and loss of soluble solids could therefore reduce the apparent gravimetric mass gain, while the panel continues to swell and lose structural integrity. Similar complications are recognized in highly hydrophilic or partly soluble bio-based systems, where water absorption alone may not fully describe dimensional degradation [7,8,9,45].

Panel density also contributed to the measured response but cannot explain the observed threshold by itself. The mean density decreased by only approximately 21 kg·m^−3^ between SCG60–ALS40 and SCG50–ALS50, whereas thickness swelling increased more than sixfold. The abrupt deterioration therefore indicates a major change in matrix continuity, water solubility, or the stability of the fiber–matrix network rather than a simple density effect.

In the preceding study, panels containing equal SCG and ALS shares exhibited water absorption values of approximately 97.9–138.6% and thickness swelling values of 22.3–48.2%, depending on the total matrix content [50]. The present SCG70–ALS30 and SCG60–ALS40 formulations showed lower water absorption than the previously investigated equal-ratio systems, supporting the beneficial effect of shifting the matrix toward SCG. Nevertheless, their dimensional behavior indicates that the material remains more suitable for dry-use applications unless the matrix is further modified, crosslinked, or hydrophobized.

### 3.3. Mechanical Properties

**Figure 4:**
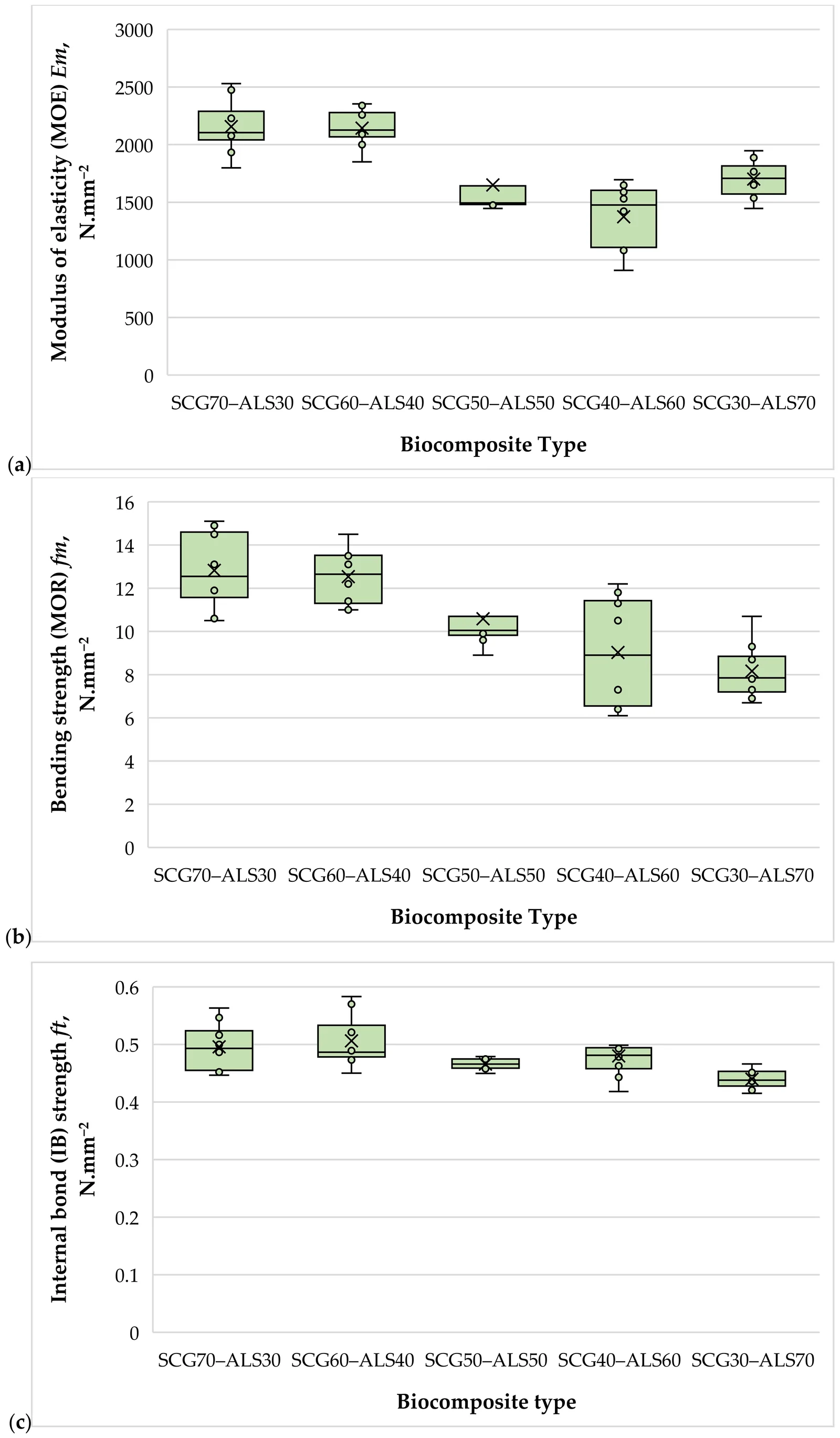
Mechanical properties of the wood-fiber biocomposites produced with different SCG:ALS ratios: (**a**) modulus of elasticity, (**b**) bending strength, and (**c**) internal bond strength. The boxes indicate the interquartile range, the horizontal line and cross within each box represent the median and arithmetic mean, respectively, and the whiskers indicate the range of non-outlying values; n=8 within-panel specimens per formulation.

The mechanical properties are presented in Figure 4. The two SCG-rich formulations exhibited the highest bending stiffness and strength. SCG70–ALS30 reached a modulus of elasticity of 2156.5 N·mm^−2^ and a bending strength of 12.83 N·mm^−2^, while SCG60–ALS40 achieved closely comparable values of 2143.1 and 12.54 N·mm^−2^, respectively.

Increasing the ALS share to 50% reduced the modulus of elasticity to 1649.9 N·mm^−2^ and the bending strength to 10.59 N·mm^−2^. The lowest modulus of elasticity was obtained for SCG40–ALS60, at 1374.3 N·mm^−2^. This formulation also showed the largest dispersion in stiffness, indicating greater within-panel heterogeneity. SCG30–ALS70 showed a partial recovery in modulus of elasticity to 1700.9 N·mm^−2^, but its bending strength decreased further to 8.16 N·mm^−2^.

The decrease in bending properties with increasing ALS can be attributed to both structural and compositional effects. The wood fibers constitute the principal load-bearing network, whereas the SCG–ALS phase transfers stress among the fibers and fills the inter-fiber spaces. Effective performance requires sufficient matrix distribution without excessive disruption of fiber continuity. Natural and self-binding lignocellulosic systems rely on intimate contact, mechanical interlocking, secondary bonding, and the presence of a sufficiently continuous reinforcing network [45,46,47].

SCG particles may contribute to stiffness as a comparatively rigid lignocellulosic filler, provided that they are adequately dispersed and integrated into the ALS-containing phase. Comparable behavior has been observed in polymer composites, where SCG addition increased stiffness at suitable concentrations but reduced strength when particle agglomeration, poor wetting, or weak interfacial adhesion became dominant [17,18,19,20,21,22,23,24,25,26]. In the present panels, the SCG-rich formulations also reached higher densities, which probably increased the number of effective contacts and reduced defect volume.

The lower and more variable mechanical performance of the ALS-rich panels may reflect greater matrix mobility during pressing, less stable structural fixation during cooling, and increased local heterogeneity. Although the total solid concentration and overall water content of the matrix suspension were maintained constant, increasing the ALS proportion altered the distribution, solubility, and plasticization behavior of the matrix during hot pressing. Previous work on the same material system showed that excessive total SCG–ALS content reduced bending properties because of difficult moisture removal, local steam pressure, and a lower relative proportion of load-bearing fibers [50]. In the present study, the total matrix content was kept constant; therefore, the observed differences are more appropriately attributed to changes in matrix composition, mobility, and interaction with the fiber network.

The internal bond results did not reveal a distinct optimum between the two SCG-rich formulations. SCG70–ALS30 and SCG60–ALS40 exhibited practically identical mean internal bond strengths of 0.509 and 0.506 N·mm^−2^, respectively. Increasing the ALS proportion to 50–70% resulted in lower values of 0.466, 0.480, and 0.440 N·mm^−2^ for SCG50–ALS50, SCG40–ALS60, and SCG30–ALS70, respectively.

The comparable internal bond performance of the 70:30 and 60:40 formulations indicates that an ALS proportion of 30–40% was sufficient to distribute the matrix over the wood-fiber and SCG surfaces and to develop through-thickness cohesion under the applied processing conditions. Increasing the ALS proportion beyond this range did not improve internal bonding. The greater water sensitivity and plasticization of the ALS-rich matrix may have offset any potential benefit arising from its improved spreading ability. This interpretation is consistent with studies showing that lignosulfonates can contribute to adhesion at suitable proportions, whereas excessive amounts of unmodified lignosulfonate may impair bond performance unless chemical modification or reactive co-binders are employed [34,35,36,37,38,39,40,41,42,43].

The preceding SCG–ALS study obtained a maximum modulus of elasticity of approximately 2707 N·mm^−2^, a bending strength of 22.55 N·mm^−2^, and an internal bond strength of approximately 0.65 N·mm^−2^ at an optimum total binder content and a fixed SCG:ALS ratio of 1:1 [50]. The lower bending properties recorded in the present work may be related to the larger matrix-to-fiber ratio, which reduced the relative contribution of the reinforcing wood-fiber network. The internal bond strengths of the present formulations remained below the maximum value reported previously, although the SCG70–ALS30 and SCG60–ALS40 panels still developed comparable and practically relevant through-thickness cohesion under the applied manufacturing conditions.

The findings demonstrate that the most favorable SCG:ALS ratio depends on the property considered. SCG70–ALS30 provided the highest bending stiffness and strength, together with the best moisture resistance. SCG60–ALS40 retained almost the same bending performance but achieved substantially higher internal bond strength. Accordingly, the interval between 60:40 and 70:30 appears to offer the most rational overall performance under the applied manufacturing conditions.

**Figure 5:**
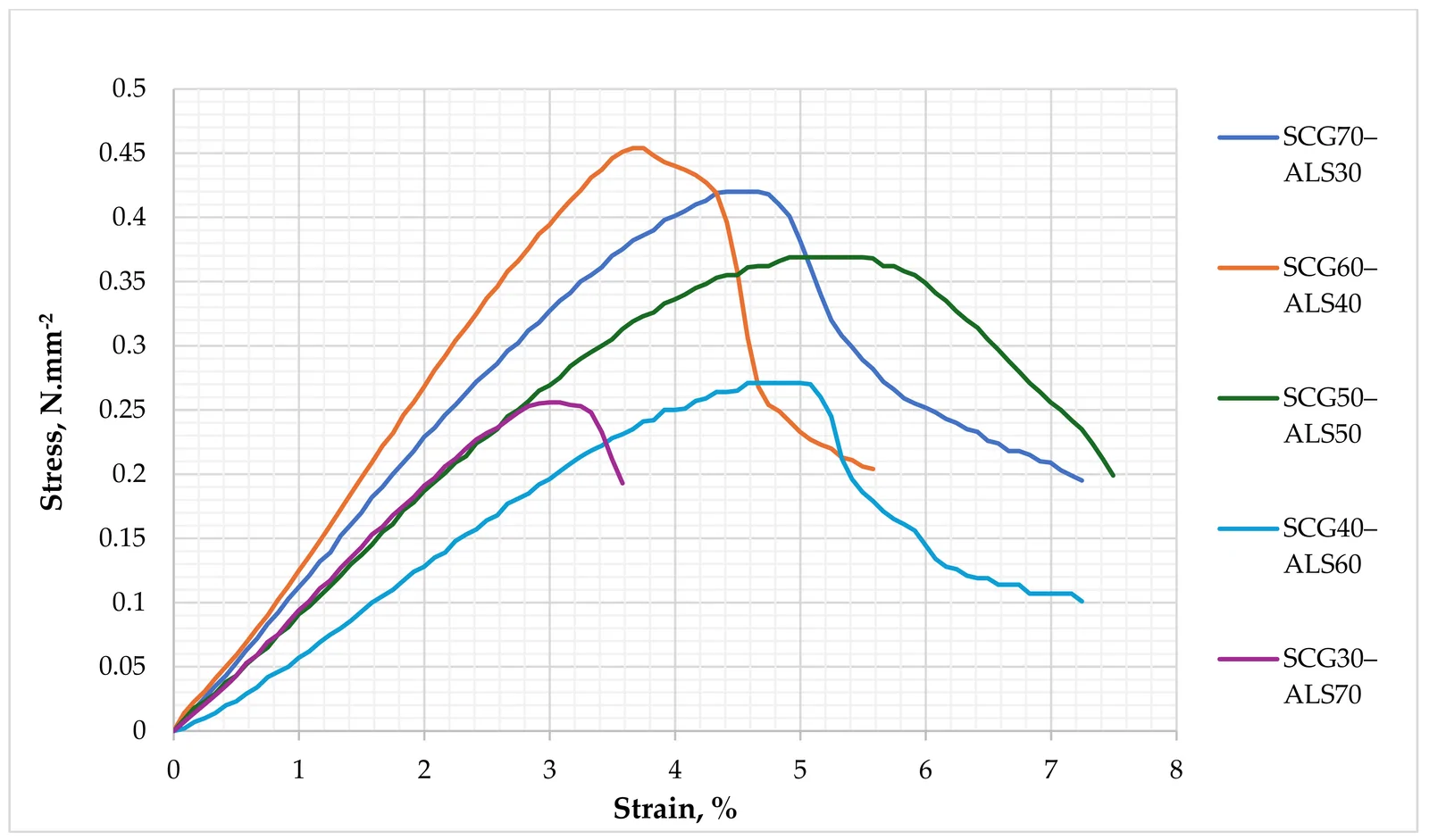
Mean flexural stress–strain curves of the wood-fiber biocomposites produced with different SCG:ALS ratios.

The differences in bending performance were further examined using the flexural stress–strain response. For each formulation, the individual curves of the eight tested specimens were interpolated to a common strain scale and averaged to provide a representative formulation-level response (Figure 5). The curves were obtained from eight within-panel specimens per formulation after interpolation of the individual curves to a common flexural-strain scale. Mean values are shown while at least six of the eight specimens contributed measured data at the corresponding strain level.

The mean flexural stress–strain curves provide additional insight into the deformation behavior of the five formulations and support the trends observed for MOE and MOR. The two SCG-rich formulations, SCG70–ALS30 and SCG60–ALS40, exhibited the steepest initial response and maintained the highest flexural stress over most of the investigated strain range. Their curves were comparatively similar during the initial and intermediate stages of loading, confirming that increasing the SCG content to 60–70% of the matrix favored both stiffness and load-bearing capacity. The SCG50–ALS50 formulation showed an intermediate response, with a lower initial slope and reduced maximum stress, consistent with the decline in its measured MOE and MOR.

A further increase in the ALS proportion resulted in a more pronounced deterioration of the flexural response. SCG40–ALS60 exhibited the lowest initial stiffness and a comparatively extended deformation response, indicating lower resistance to bending but a more gradual reduction in load-bearing capacity in part of the tested strain range. SCG30–ALS70 also showed reduced flexural strength despite the partial recovery in stiffness observed from the MOE results. Overall, the stress–strain behavior confirms a progressive change in the flexural response as the matrix composition shifts from SCG-rich toward ALS-rich formulations. This trend is consistent with the proposed composition-dependent differences in panel consolidation, matrix distribution, and stress transfer within the fiber–matrix structure. The maxima of the averaged curves should not be interpreted as the arithmetic mean MOR values because the individual specimens reached their maximum flexural stress at different strain levels.

### 3.4. Thermal Behavior of the SCG–ALS Matrix Formulations

The thermal measurements were performed on the SCG–ALS matrix formulations before their incorporation into the wood fibers. The results therefore characterize the early thermal response of the matrix mixtures and should not be interpreted as the thermal behavior of the finished panels.

**Figure 6:**
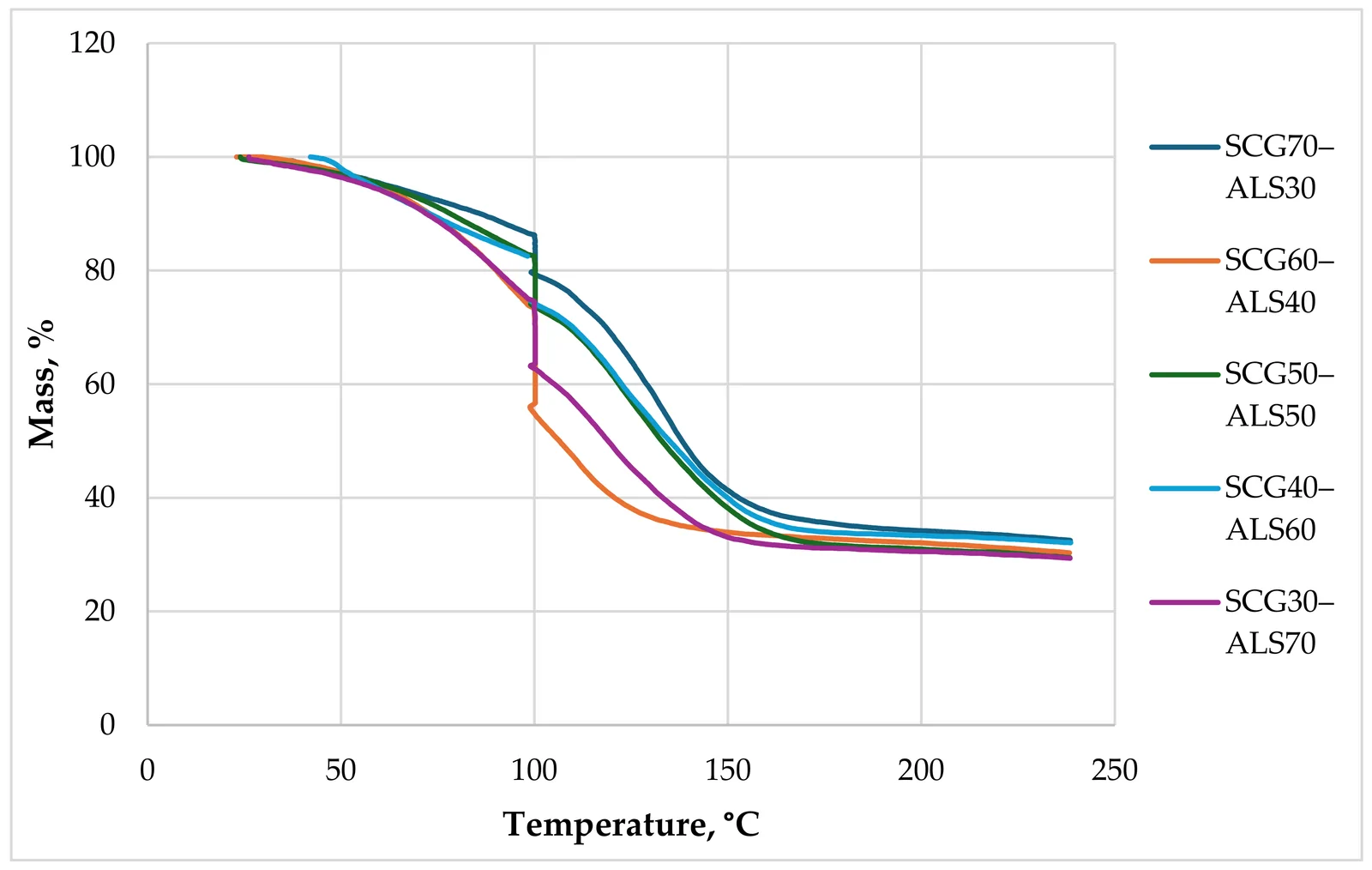
TG curves of the SCG–ALS matrix formulations with different SCG:ALS ratios. The samples were heated in air to approximately 100 °C, held isothermally for about 4.9 min, and subsequently heated to approximately 238 °C. The near-vertical segment at 100 °C represents mass change during the isothermal holding stage and should not be interpreted as a discrete temperature-induced transition.

Figure 6 presents the TG curves of the five formulations. A substantial mass decrease occurred during heating to 100 °C and the subsequent isothermal stage. Depending on the formulation, the mass loss by the end of the holding period ranged from 19.87 to 43.03%.

This initial loss can be attributed mainly to free and physically associated water, together with volatile low-molecular compounds present in SCGs and the commercial ALS solution. SCGs contain residual oils, carbohydrates, proteins, phenolic substances, and other extractives whose proportions depend on the coffee origin, roasting, and brewing process [10,11,12,13,14]. Commercial lignosulfonates similarly contain water, low-molecular lignin fragments, residual carbohydrates, and inorganic constituents [27,28,29,30,31,32,33,34,35,36,37]. The absence of a monotonic relationship between the SCG:ALS ratio and the initial mass loss suggests that water distribution and small-sample heterogeneity influenced the results in addition to nominal composition.

**Figure 7:**
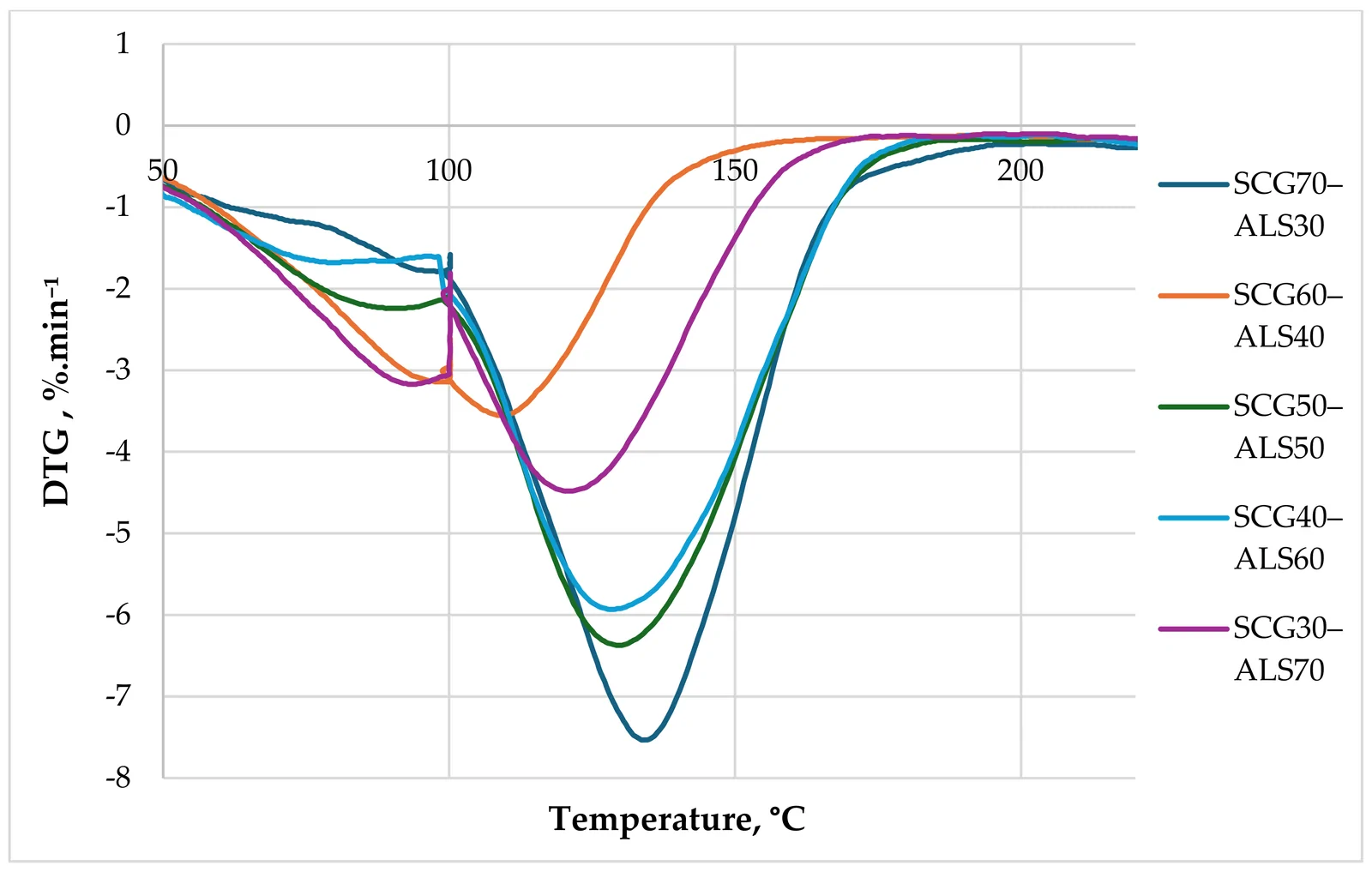
DTG curves of the SCG–ALS matrix formulations with different SCG:ALS ratios. The curves represent the derivative mass-loss rate during the applied heating program. The discontinuity around 100 °C is associated with the isothermal holding stage and the transition between the dynamic heating segments.

The DTG curves are shown in Figure 7. The temperatures of maximum mass-loss rate, $T_{DTG,max}$, varied from approximately 109 to 134 °C, indicating that the matrix ratio modified the position of the dominant early mass-loss event.

The principal mass-loss event occurred at the lowest temperature for SCG40–ALS60 and at the highest temperature for SCG30–ALS70. The variation was not monotonic, demonstrating that the thermal response did not depend simply on the concentration of one constituent. Instead, it reflected overlapping water release, volatilization, decomposition of low-molecular compounds, and early thermally induced transformations within the lignin- and carbohydrate-containing matrix.

The thermal decomposition of lignocellulosic materials generally involves overlapping transformations of hemicelluloses, cellulose, lignin, and extractives rather than a single discrete reaction [48,49]. Lignin-derived materials typically show broad thermal responses because of their heterogeneous aromatic structure and wide molecular-mass distribution [27,28,29,30]. The presence of sulfonate and ammonium functionalities in ALS may further modify the release of volatile species and the formation of thermally stable residues, as shown in Table 1.

**Figure 8:**
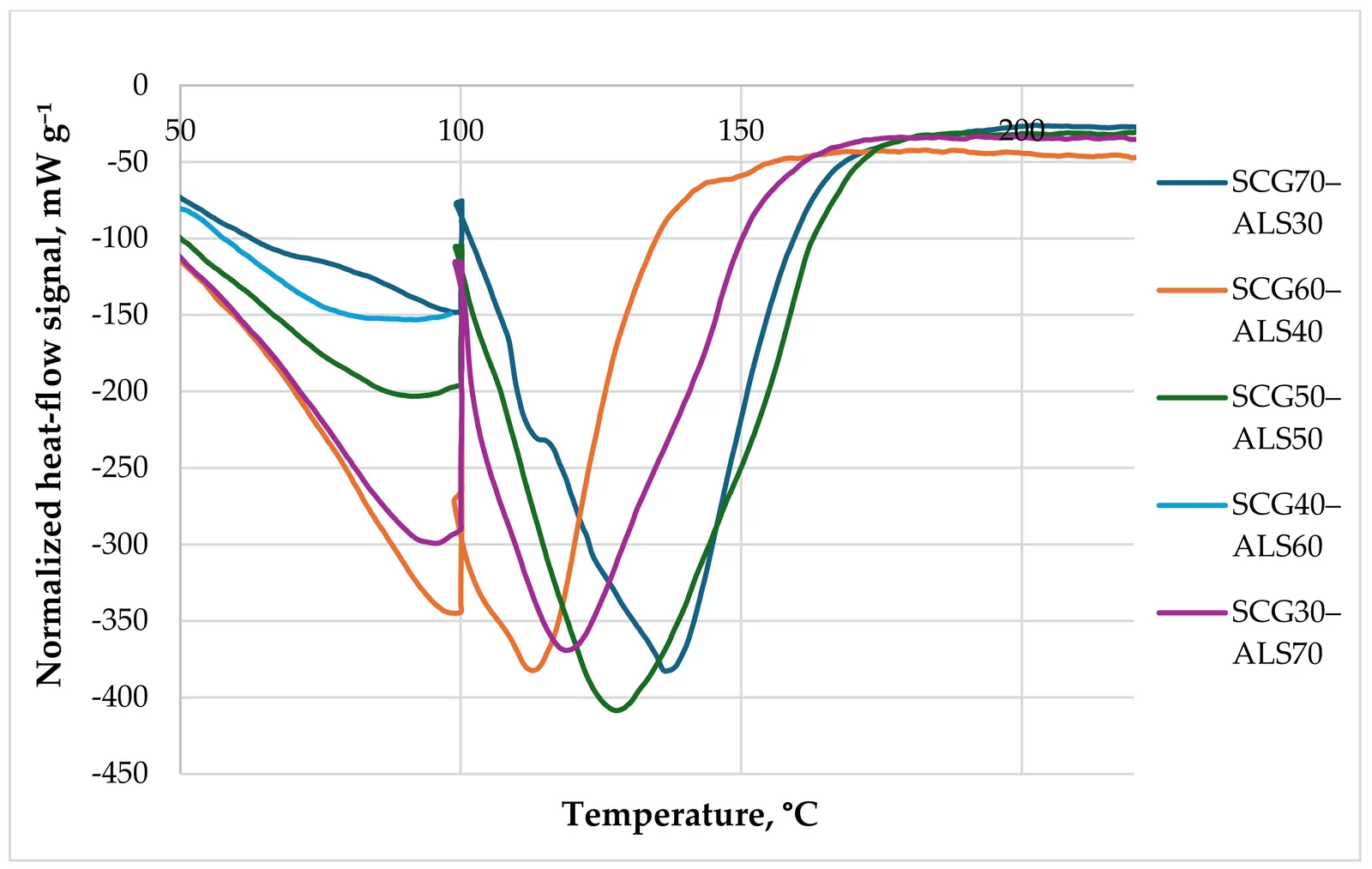
Baseline-corrected heat-flow curves of the SCG–ALS matrix formulations, normalized to the sample mass recorded at the end of the isothermal holding stage at approximately 100 °C. The discontinuity around 100 °C results from the isothermal holding stage and the transition between the dynamic heating segments.

The baseline-corrected heat-flow curves, normalized to the sample mass recorded at the end of the 100 °C isothermal holding stage, are presented in Figure 8.

The principal heat-flow minima occurred between approximately 113 and 136 °C and closely corresponded to the main DTG events. This agreement indicates that the detected thermal events were accompanied by measurable mass loss. They should therefore not be assigned directly to a pure glass transition, melting point, or another exclusively physical phase transition.

A glass transition normally appears mainly as a change in heat capacity and a shift in the DSC baseline, without substantial simultaneous mass loss. In the present case, the correspondence between the heat-flow and DTG responses indicates a combined process involving residual bound water, volatile SCG components, possible ammonia-containing species from ALS, and early chemical transformations of the matrix constituents.

SCG30–ALS70 exhibited the highest principal-event temperature, whereas SCG40–ALS60 showed the lowest. Because the trend was not monotonic, the values should not be used to claim that increasing ALS systematically improved or reduced thermal stability. Rather, the SCG:ALS ratio altered the relative contribution and overlap of several thermal processes.

The remaining mass at approximately 238 °C varied within the comparatively narrow interval of 29.39–32.53%. These values do not represent final char or ash yield because the measurement ended before the principal decomposition of cellulose and the broader degradation of lignin were completed [48,49]. The thermal analysis should therefore be interpreted as a comparative assessment of the early and processing-relevant thermal behavior of the matrix.

The current analysis differs fundamentally from the thermal evaluation reported in the preceding study [50]. In that work, complete wood-fiber panels were analyzed over a considerably wider temperature range, and major thermal events were recorded at approximately 270–301 °C, with final combustion-related processes extending to approximately 595–625 °C. In the present work, only the SCG–ALS matrix was examined, and the analysis ended at approximately 238 °C. The two datasets are therefore complementary: the previous results characterize the broader degradation behavior of the finished composites, whereas the current results describe the early thermal response of the binder formulations.

### 3.5. Overall Performance and Study Limitations

The combined results demonstrated that the SCG:ALS ratio is an important formulation parameter influencing panel consolidation and overall performance. Within the investigated compositional range, increasing the SCG proportion was generally associated with higher achieved density, improved water resistance, and enhanced bending stiffness and strength. At the same time, the presence of ALS likely supported matrix dispersion and internal cohesion, although its essential role cannot be confirmed without an ALS-free control. Overall, the findings suggest that panel performance depends on achieving an appropriate balance between the water-soluble lignosulfonate-derived binding phase and the particulate coffee-derived phase, rather than on a simple linear advantage associated with increasing either constituent.

The SCG70–ALS30 panel exhibited the lowest water absorption and thickness swelling values, together with the highest modulus of elasticity and bending strength among the investigated formulations. The SCG60–ALS40 panel showed closely comparable bending properties, while both SCG-rich formulations achieved nearly identical internal bond strengths. These findings identify the range containing 30–40% ALS as a promising compositional region for further optimization and validation using independently replicated panels. In contrast, formulations containing 50% or more ALS displayed pronounced dimensional instability and generally inferior mechanical performance under the applied manufacturing conditions.

These findings contribute to addressing the research gap identified in the preceding investigations, in which the total SCG–ALS loading was varied while the two constituents were maintained at an equal ratio [50,51]. By keeping the nominal total matrix content constant and varying the internal SCG:ALS composition, the present study provides preliminary evidence that the relative proportions of the two matrix constituents are associated with substantial differences in panel performance. However, because achieved panel density also varied among the formulations, the effects of matrix composition and densification could not be completely separated.

Several limitations must therefore be acknowledged. Only one laboratory panel was manufactured for each formulation, and the eight test specimens constituted within-panel subsamples rather than independent panel-production replicates. Consequently, the reported variability describes local heterogeneity within each panel and does not support population-level statistical inference or definitive attribution of the observed differences to formulation alone. The results should therefore be interpreted as exploratory, formulation-associated trends requiring validation through independently replicated panel production and improved density control.

Second, the achieved panel density varied considerably with matrix composition. Because density directly influences porosity, water uptake, dimensional stability, stiffness, strength, and internal bond performance, the respective contributions of matrix composition and panel consolidation could not be fully separated. Future studies should therefore include independently replicated panels and tighter control of the achieved density across all formulations, preferably supplemented by density-profile analysis.

Third, the ALS-rich panels exhibited exceptionally high thickness swelling. Because ALS is water-soluble, leaching of soluble matrix constituents during immersion may have affected the gravimetrically determined water absorption values. Analysis of the immersion water, quantification of dissolved solids, and redrying of the specimens to constant mass would help distinguish actual water uptake from material loss.

Fourth, only one thermal-analysis run was performed for each formulation. Moreover, pure SCGs, pure ALS, wood fibers, and finished-panel controls were not examined using the same temperature program. Consequently, the attribution of individual thermal events to specific matrix constituents or interactions remains provisional. The maximum temperature of approximately 238 °C was also insufficient to characterize complete thermal decomposition and final residue formation. Future investigations should employ replicated measurements, appropriate constituent and composite controls, and an extended temperature range.

The present study was deliberately focused on the direct macroscopic response of the panels and on the early thermal behavior of the SCG–ALS matrix. Density, water absorption, thickness swelling, bending properties, internal bond strength, and simultaneous thermal analysis provide an efficient and application-oriented basis for identifying the influence of matrix composition and for selecting the most promising formulation range. Restricting the current work to these directly comparable performance indicators also allowed the SCG:ALS ratio to be evaluated without combining the principal composition–property analysis with several separate mechanistic investigations. Microscopy, FTIR spectroscopy, density-profile analysis, determination of extractable or leachable matter, and quantitative evaluation of failure modes are therefore proposed as targeted follow-up methods. These techniques should be applied primarily to the promising SCG70–ALS30 and SCG60–ALS40 formulations, as well as to the transition region around 50% ALS, in order to clarify matrix distribution, hydrogen-bonding interactions, local porosity, soluble-material migration, and the mechanisms governing mechanical failure.

Despite these limitations, this study provides a clear composition–property relationship for the SCG–ALS matrix. Under the applied laboratory conditions, formulations containing 60–70% SCG and 30–40% ALS provided the most favorable balance among dimensional stability, flexural performance, and internal cohesion.

## 4. Conclusions

This study demonstrated that the internal SCG:ALS ratio is a key formulation parameter governing the performance of wood-fiber biocomposites manufactured at a constant matrix content and under identical processing conditions. Within the investigated range, increasing the proportion of spent coffee grounds was generally associated with higher achieved panel density, improved dimensional stability, and enhanced bending performance. In contrast, formulations containing 50% or more ammonium lignosulfonate exhibited pronounced water sensitivity and generally lower mechanical performance.

The SCG70–ALS30 panel exhibited the lowest water absorption and thickness swelling, together with the highest modulus of elasticity and bending strength. The SCG60–ALS40 panel achieved closely comparable bending performance, while both SCG-rich panels showed nearly identical internal bond strengths. These observations identify SCG:ALS ratios between 70:30 and 60:40 as the most promising compositional range under the applied manufacturing conditions. Within this range, SCG contributes to improved packing, structural stability, and flexural performance, while an ALS proportion of 30–40% appears sufficient to support matrix distribution and through-thickness cohesion under the applied manufacturing conditions.

The principal novelty and scientific significance of this study lie in the systematic isolation of the SCG:ALS ratio as an independent formulation variable while maintaining the total matrix content, suspension concentration, nominal panel density, and pressing conditions constant. In contrast to previous investigations focused mainly on the total amount of an equimass SCG–ALS matrix, the present results identify a critical transition between 40 and 50% ALS, above which dimensional stability decreases sharply. This study therefore establishes that the internal balance between the particulate SCG fraction and the water-soluble ALS phase is at least as important as the total amount of matrix incorporated into the composite. This finding is scientifically significant because it identifies the internal matrix composition as a distinct design parameter that should be considered independently from the total binder content when optimizing SCG–ALS wood-fiber biocomposites.

The simultaneous thermal analysis further showed that changing the SCG:ALS ratio modified water release, mass-loss behavior, and the position of the principal early heat-flow event. The correspondence between the DTG and heat-flow responses indicates that these events resulted from overlapping volatilization and early thermal transformations rather than from a single phase transition. These findings complement the physical and mechanical results by demonstrating that matrix composition also controls its processing-relevant thermal response. The present thermal analysis was intentionally limited to the moisture-containing SCG–ALS matrix and to the early temperature range relevant to hot pressing; therefore, it does not represent a complete dry-state thermal-degradation characterization of the matrix or the finished biocomposites. Future work will include separate moisture determination and extended TGA–DSC measurements of fully dried SCG–ALS formulations and finished biocomposite panels over a substantially wider temperature range in order to establish their complete thermal stability and decomposition behavior.

Overall, this study defines a promising formulation window for the further development of formaldehyde-free wood-fiber biocomposites based on two renewable secondary resources. SCG70–ALS30 provided the most favorable overall combination of dimensional stability and bending performance, while SCG60–ALS40 achieved closely comparable flexural and internal bond properties. These formulations therefore provide a rational basis for replicated validation, further optimization, and dedicated structural, microscopic, and physicochemical characterization aimed at verifying the proposed interfacial and adhesion mechanisms, as well as for the development of panels intended primarily for dry-use applications. From a practical perspective, defining this formulation window provides a more rational basis for future material optimization and reduces reliance on empirical selection of SCG–ALS proportions. The proposed material concept advances the circular bioeconomy by transforming spent coffee grounds and lignosulfonates into value-added composite matrices, extending their material life while reducing waste and dependence on fossil-derived adhesives.

## Figures and Tables

**Table 1:** Selected thermal parameters of the SCG–ALS matrix formulations.

SCG:ALS Ratio	Mass Loss by the End of the 100 °C Hold, %	(Temperature of Maximum Mass-Loss Rate *T*_DTG,max_), °C	Principal Corrected Heat-Flow Minimum, °C	Remaining Mass at Approximately 238 °C, %
70:30	36.27	120.87	119.17	29.39
60:40	24.69	128.42	125.83	32.10
50:50	25.33	129.73	128.17	29.49
40:60	43.03	109.15	113.23	30.33
30:70	19.87	134.10	136.40	32.53

## Data Availability

The original contributions presented in this study are included in this article. Further inquiries can be directed to the corresponding authors.

## References

[1] Calvez I., Garcia R., Koubaa A., Landry V., Cloutier A. (2024). Recent Advances in Bio-Based Adhesives and Formaldehyde-Free Technologies for Wood-Based Panel Manufacturing. Curr. For. Rep..

[2] Garcia R., Calvez I., Koubaa A., Landry V., Cloutier A. (2024). Sustainability, Circularity, and Innovation in Wood-Based Panel Manufacturing in the 2020s: Opportunities and Challenges. Curr. For. Rep..

[3] Arias A., González-Rodríguez S., Barros M.V., Salvador R., de Francisco A.C., Piekarski C.M., Moreira M.T. (2021). Recent Developments in Bio-Based Adhesives from Renewable Natural Resources. J. Clean. Prod..

[4] Pizzi A., Papadopoulos A.N., Policardi F. (2020). Wood Composites and Their Polymer Binders. Polymers.

[5] Dunky M. (1998). Urea–Formaldehyde (UF) Adhesive Resins for Wood. Int. J. Adhes. Adhes..

[6] Krišťák Ľ., Antov P., Bekhta P., Lubis M.A.R., Iswanto A.H., Réh R., Sedliačik J., Savov V., Taghiyari H.R., Papadopoulos A.N. (2023). Recent Progress in Ultra-Low Formaldehyde Emitting Adhesive Systems and Formaldehyde Scavengers in Wood-Based Panels: A Review. Wood Mater. Sci. Eng..

[7] Hemmilä V., Adamopoulos S., Karlsson O., Kumar A. (2017). Development of Sustainable Bio-Adhesives for Engineered Wood Panels—A Review. RSC Adv..

[8] Ferdosian F., Pan Z., Gao G., Zhao B. (2017). Bio-Based Adhesives and Evaluation for Wood Composites Application. Polymers.

[9] Hussin M.H., Abd Latif N.H., Hamidon T.S., Idris N.N., Hashim R., Appaturi J.N., Brosse N., Ziegler-Devin I., Chrusiel L., Fatriasari W. (2022). Latest Advancements in High-Performance Bio-Based Wood Adhesives: A Critical Review. J. Mater. Res. Technol..

[10] McNutt J., He Q.S. (2019). Spent Coffee Grounds: A Review on Current Utilization. J. Ind. Eng. Chem..

[11] Mussatto S.I., Machado E.M.S., Martins S., Teixeira J.A. (2011). Production, Composition, and Application of Coffee and Its Industrial Residues. Food Bioprocess Technol..

[12] Campos-Vega R., Loarca-Piña G., Vergara-Castañeda H.A., Oomah B.D. (2015). Spent Coffee Grounds: A Review on Current Research and Future Prospects. Trends Food Sci. Technol..

[13] Ballesteros L.F., Teixeira J.A., Mussatto S.I. (2014). Chemical, Functional, and Structural Properties of Spent Coffee Grounds and Coffee Silverskin. Food Bioprocess Technol..

[14] Caetano N.S., Silva V.F.M., Melo A.C., Martins A.A., Mata T.M. (2014). Spent Coffee Grounds for Biodiesel Production and Other Applications. Clean Technol. Environ. Policy.

[15] Saberian M., Li J., Donnoli A., Bondarenko E., Oliva P., Gill B., Lockrey S., Siddique R. (2021). Recycling of Spent Coffee Grounds in Construction Materials: A Review. J. Clean. Prod..

[16] Ahmed H., Abolore R.S., Jaiswal S., Jaiswal A.K. (2024). Toward Circular Economy: Potentials of Spent Coffee Grounds in Bioproducts and Chemical Production. Biomass.

[17] Gaidukova G., Gaidukovs S., Grase L., Platnieks O., Sereda A., Andriotis E.G., Antonova M., Yamaguchi K., Enachescu M. (2021). Spent Coffee Waste as a Renewable Source for the Production of Polymer Composite Fillers. RSC Adv..

[18] Essabir H., Raji M., Ait Laaziz S., Rodrigue D., Bouhfid R., Qaiss A.E.K. (2018). Thermo-Mechanical Performances of Polypropylene Biocomposites Based on Untreated, Treated and Compatibilized Spent Coffee Grounds. Compos. Part B Eng..

[19] Lage-Rivera S., Ares-Pernas A.I., Dopico-García S., Covas J., Abad M.J. (2024). Comparing Lignin and Spent Coffee Grounds as Bio-Fillers in PLA 3D-Printable Filaments. Polym. Compos..

[20] Yu W., Yuan T., Yao Y., Deng Y., Wang X. (2023). PLA/Coffee Grounds Composite for 3D Printing and Its Properties. Forests.

[21] Massijaya S.Y., Lubis M.A.R., Nissa R.C., Nurhamiyah Y., Nugroho P., Antov P., Lee S.-H., Papadopoulos A.N., Kusumah S.S., Karlinasari L. (2023). Utilization of Spent Coffee Grounds as a Sustainable Resource for the Synthesis of Bioplastic Composites with Polylactic Acid, Starch, and Sucrose. J. Compos. Sci..

[22] Bejenari V., Danu M., Ipate A.-M., Zaltariov M.-F., Rusu D., Lisa G. (2024). Composite Materials Based on Spent Coffee Grounds and Paper Pulp. J. Compos. Sci..

[23] Janowski G., Frącz W., Bąk Ł., Sikora J.W., Tomczyk A., Mrówka-Nowotnik G., Mossety-Leszczak B. (2025). Effect of Coffee Grounds Content on Properties of PHBV Biocomposites Compared to Similar Composites with Other Fillers. Polymers.

[24] Waisarikit A., Suadaung N., Khantho B., Hadad B., Ross G.M., Topham P.D., Ross S., Mahasaranon S. (2025). Extracted Spent Coffee Grounds as a Performance-Enhancing Additive for Poly(Lactic Acid) Biodegradable Nursery Bags in Agriculture. Polymers.

[25] Xiao S., Zhu X., Zhang Y., Guo Y., Wei W., Guo Q. (2022). The Biocomposites Properties of Compounded Polylactic Acid with Untreated and Treated Spent Coffee Grounds. J. Appl. Polym. Sci..

[26] Mäder G., Rüegg N., Tschichold T., Yildirim S. (2025). Utilizing Spent Coffee Grounds as Sustainable Fillers in Biopolymer Composites: Influence of Particle Size and Content. Sustain. Food Technol..

[27] Kropat M., Liao M., Park H., Salem K.S., Johnson S., Argyropoulos D.S. (2021). A Perspective of Lignin Processing and Utilization Technologies for Composites and Plastics with Emphasis on Technical and Market Trends. BioResources.

[28] Tanase-Opedal M., Espinosa E., Rodríguez A., Chinga-Carrasco G. (2019). Lignin: A Biopolymer from Forestry Biomass for Biocomposites and 3D Printing. Materials.

[29] Belgacem M.N., Gandini A. (2008). Lignins as Components in Polymers. Prog. Polym. Sci..

[30] Laurichesse S., Avérous L. (2014). Chemical Modification of Lignins: Towards Biobased Polymers. Prog. Polym. Sci..

[31] Pizzi A. (2006). Recent Developments in Eco-Efficient Bio-Based Adhesives for Wood Bonding: Opportunities and Issues. J. Adhes. Sci. Technol..

[32] Ghaffar S.H., Fan M. (2014). Lignin in Straw and Its Applications as an Adhesive. Int. J. Adhes. Adhes..

[33] Gosselink R.J.A., van Dam J.E.G., de Jong E., Gellerstedt G., Scott E.L., Sanders J.P.M. (2011). Effect of Periodate on Lignin for Wood Adhesive Application. Holzforschung.

[34] Hemmilä V., Adamopoulos S., Hosseinpourpia R., Ahmed S.A. (2019). Ammonium Lignosulfonate Adhesives for Particleboards with pMDI and Furfuryl Alcohol as Crosslinkers. Polymers.

[35] Bekhta P., Noshchenko G., Réh R., Krišťák Ľ., Sedliačik J., Antov P., Mirski R., Savov V. (2021). Properties of Eco-Friendly Particleboards Bonded with Lignosulfonate–Urea–Formaldehyde Adhesives and pMDI as a Crosslinker. Materials.

[36] Antov P., Mantanis G.I., Savov V. (2020). Development of Wood Composites from Recycled Fibres Bonded with Magnesium Lignosulfonate. Forests.

[37] Lourenço A., Gominho J., Marques A.V., Pereira H. (2013). Comparison of Lignosulfonates and Kraft Lignin in the Production of Fiberboards. Ind. Crops Prod..

[38] Gao S., Cheng Z., Zhou X., Liu Y., Chen R., Wang J., Wang C., Chu F., Xu F., Zhang D. (2020). Unexpected Role of Amphiphilic Lignosulfonate to Improve the Storage Stability of Urea–Formaldehyde Resin and Its Application as Adhesives. Int. J. Biol. Macromol..

[39] Balea A., Paul A., Blanco S., Pecha J., Mensah R.A., Blanco A., Negro C. (2021). Mechanical Properties and Formaldehyde Release of Particleboard Made with Different Lignin-Based Adhesives. Appl. Sci..

[40] Balea G., Lunguleasa A., Zeleniuc O., Coșereanu C. (2022). Three Adhesive Recipes Based on Magnesium Lignosulfonate, Used to Manufacture Particleboards with Low Formaldehyde Emissions and Good Mechanical Properties. Forests.

[41] Gonçalves S., Paiva N.T., Martins J., Carvalho L.H., Magalhães F.D. (2024). Fast-Curing Three-Layer Particleboards with Lignosulfonate and pMDI Adhesives. Forests.

[42] Gonçalves S., Paiva N.T., Martins J., Magalhães F.D., Carvalho L.H. (2024). Effect of Lignosulphonates on the Moisture Resistance of Phenol–Formaldehyde Resins for Exterior Plywood. Materials.

[43] Lytvyn I., Bekhta N., Kusniak I., Bekhta P., Sedliačik J. (2025). Properties of Particleboards with Partial Replacement of MUF Resin by Sodium and Magnesium Lignosulfonates. Wood Res..

[44] Kubo S., Kadla J.F. (2005). Hydrogen Bonding in Lignin: A Fourier Transform Infrared Model Compound Study. Biomacromolecules.

[45] Hubbe M.A., Pizzi A., Zhang H., Halis R. (2018). Critical Links Governing Performance of Self-Binding and Natural Binders for Hot-Pressed Reconstituted Lignocellulosic Board without Added Formaldehyde: A Review. BioResources.

[46] Pintiaux T., Viet D., Vandenbossche V., Rigal L., Rouilly A. (2015). Binderless Materials Obtained by Thermo-Compressive Processing of Lignocellulosic Fibers: A Comprehensive Review. BioResources.

[47] Oliaei E., Berthold F., Berglund L.A., Lindström T. (2021). Eco-Friendly High-Strength Composites Based on Hot-Pressed Lignocellulose Microfibrils or Fibers. ACS Sustain. Chem. Eng..

[48] Yang H., Yan R., Chen H., Lee D.H., Zheng C. (2007). Characteristics of Hemicellulose, Cellulose and Lignin Pyrolysis. Fuel.

[49] Kunaver M., Jasiukaitytė E., Čuk N., Medved S. (2010). The Fast Pyrolysis of Lignocellulosic Biomass: The Effect of Lignin Content. Bioresour. Technol..

[50] Savov V., Antov P., Kostadinova-Slaveva A., Yusein J., Dudeva V., Todorova E., Petrin S. (2025). Development and Characterization of Sustainable Biocomposites from Wood Fibers, Spent Coffee Grounds, and Ammonium Lignosulfonate. Polymers.

[51] Kostadinova-Slaveva A., Savov V., Antov P., Malcheva B., Todorova E., Yusein J., Dudeva V., Ivanov G. (2026). Aesthetic Profiling and Exploratory Composting Screening of Wood-Fiber Biocomposites Bonded with Spent Coffee Grounds and Ammonium Lignosulfonate. Materials.

[52] Madyaratri E.W., Ridho M.R., Aristri M.A., Lubis M.A.R., Iswanto A.H., Nawawi D.S., Antov P., Krišťák Ľ., Majlingová A., Fatriasari W. (2022). Recent Advances in the Development of Fire-Resistant Biocomposites—A Review. Polymers.

[53] (1994). Wood-Based Panels—Sampling, Cutting and Inspection—Part 1: Sampling and Cutting of Test Pieces and Expression of Test Results.

[54] (2012). Wood-Based Panels—Determination of Dimensions of Test Pieces.

[55] (1993). Wood-Based Panels—Determination of Density.

[56] (1993). Particleboards and Fibreboards—Determination of Swelling in Thickness after Immersion in Water.

[57] (1993). Wood-Based Panels—Determination of Modulus of Elasticity in Bending and of Bending Strength.

[58] (1993). Particleboards and Fibreboards—Determination of Tensile Strength Perpendicular to the Plane of the Board.

